# Acute fibrinous and organizing pneumonia after lung transplantation: A case report of treatment with infliximab and tocilizumab and literature review

**DOI:** 10.1016/j.rmcr.2024.102159

**Published:** 2024-12-27

**Authors:** Christophe Abellan, Foteini Ioakeim, Alessio Casutt, Benoit Lechartier, Zisis Balmpouzis, Samuel Rotman, Leslie Noirez, Isabelle Rochat, John-David Aubert, Angela Koutsokera

**Affiliations:** a Service of Internal Medicine, Lausanne University Hospital and University of Lausanne, Lausanne, Switzerland; b Division of Pulmonology, Dept of Medicine, Lausanne University Hospital and University of Lausanne, Lausanne, Switzerland; c Service of Clinical Pathology, Lausanne University Hospital and University of Lausanne, Lausanne, Switzerland; d Adult Cystic Fibrosis and CFTR-related disorders Center, Division of Pulmonology, Dept of Medicine, Lausanne University Hospital and University of Lausanne, Switzerland; e Lung Transplantation Center, Division of Pulmonology, Dept of Medicine, Lausanne University Hospital and University of Lausanne, Switzerland; f Pediatric Pulmonology Unit, Lausanne University Hospital and University of Lausanne, Lausanne, Switzerland; g Division of Pulmonary Medicine, Department of Medicine, Ospedale Regionale di Lugano, EOC, Lugano, Switzerland; h Faculty of Biomedical sciences, Università della Svizzera Italiana (USI), Lugano, Switzerland; i Service of Intensive Care Medicine, Lausanne University Hospital and University of Lausanne, Lausanne, Switzerland

## Abstract

**Introduction:**

Acute fibrinous and organizing pneumonia (AFOP) is a severe form of acute lung injury which can occur after lung transplantation. Treatment is empiric, based on immunosuppressive regimens and the mortality rate is very high.

**Case presentation:**

We report the case of a young lung transplant (LT) recipient who developed AFOP following a respiratory viral infection while on suboptimal maintenance immunosuppression due to adherence issues. Diagnosis was confirmed by cryobiopsies showing intra-alveolar fibrin balls. Despite high dose systemic corticosteroids, the patient developed severe respiratory failure requiring mechanical ventilation. IV infliximab and tocilizumab were administered. The patient was extubated 11 days later and discharged to home 42 days after intubation with 1L/min O2. She developed severe pleuritic pain needing opioid treatment and died 4 months later.

**Conclusion:**

While high-dose systemic corticosteroids remain the first line of treatment, the use of anti TNF-α has shown promising results in case reports. Furthermore, we propose prompt realization of a cytokine panel analysis in both blood and bronchoalveolar lavage to better guide the adjuvant administration of a targeted anti-inflammatory therapy.

## Highlights

• The use of anti TNF-α has shown promising results.
• Cytokine panel analysis in both blood and bronchoalveolar lavage should be used.
• Treatment with targeted anti-inflammatory therapy seems necessary.

## 1 Introduction

Acute fibrinous and organizing pneumonia (AFOP) was firstly described by *Beasley et* al. in 2002 [1]. It is characterized by specific histopathological findings, the main feature being the presence of fibrin in alveolar spaces (“fibrin balls”). This histopathological pattern has been documented in several patients, including LT recipients, presenting rapidly progressive acute lung injury. Several factors have been associated with AFOP development however, clear evidence of causality is lacking.

While the exact incidence and prevalence of AFOP are unknown, it is a very rare entity. In LT recipients, less than a hundred cases have been reported in the literature. Treatment is empiric, based on immunosuppressive regimens and the mortality rate is very high exceeding 90 % (median survival of 101 days) [2].

## 2 Case report

We report the case of an 18-year-old female patient known for cystic fibrosis F508del homozygous who received a bilateral lung transplantation at the age of 12. The patient had no history of allograft rejection (cellular or antibody-mediated), anastomotic lesions or recurring infection. *Staphylococcus aureus* was identified in several ENT samplings. She had severe malnutrition with a BMI of 16.2 kg/m2 and a percutaneous endoscopic gastrostomy tube that was not being used for enteral feeding. Pediatric follow-up was marked with a history of inconstant adherence to immunosuppressive therapy, needing high doses of oral sustained release tacrolimus (Advagraf®, Astellas) to obtain adequate trough levels.

On the first consultation in our adult outpatient clinic, the patient had a stable respiratory situation with a stage 2 dyspnea on the modified Medical Research Council (mMRC) scale. She reported no recent history of cough, sputum production or hemoptysis. Pulmonary auscultation showed normal breath sounds and SpO2 was at 97 % on ambient air. Pulmonary function tests showed a forced expiratory volume in the first second (FEV1) of 2.16 L (72 % predicted value [PV]), a forced vital capacity (FVC) of 2.43 L (72 % PV) and a FEV1/FVC ratio of 0.89.

**Fig. 1:**
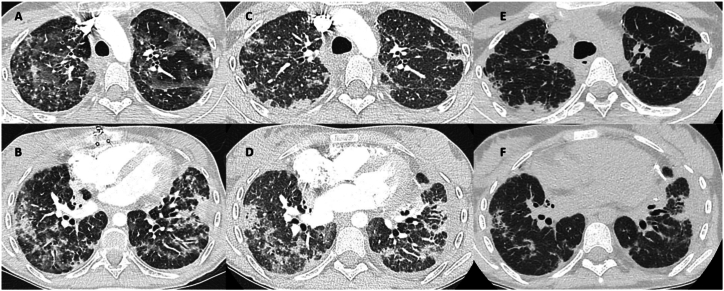
**Evolution of radiologic findings.** Panels A and B: Panlobar multifocal ground glass opacities bilaterally and peribronchial condensations with a predominance in the lower lobes. Panels C and D: Resolution of apical ground glass opacities. Panels E and F: Near complete resolution of ground glass opacities with stabilization of peribronchial condensations.

Three months later, the patient presented with rapidly worsening dyspnea, dry cough and symptoms of upper respiratory tract infection. A nasopharyngeal swab (NPS) RT-PCR showed the presence of Adenovirus and Rhinovirus (200 copies/ml and 1′570′0000 copies/ml respectively). PCR for SARS-CoV-2 was negative. Thoracic radiography did not display any signs of infection and supportive therapy was decided with a closer follow-up. Due to lack of clinical improvement and development of an inflammatory syndrome, antibiotic therapy with Levofloxacine (500 mg/day for 10 days) and Minocycline (100 mg twice daily for 4 days) was initiated. Spirometry showed a total loss of 1.14 L of FEV1, reaching 1.02 L (34 % predicted value and 47 % of her best FEV1). A chest CT scan showed panlobar multifocal bilateral ground glass opacities and peribronchial condensations with lower lobe predominance (Fig. 1A and B).

**Fig. 2:**
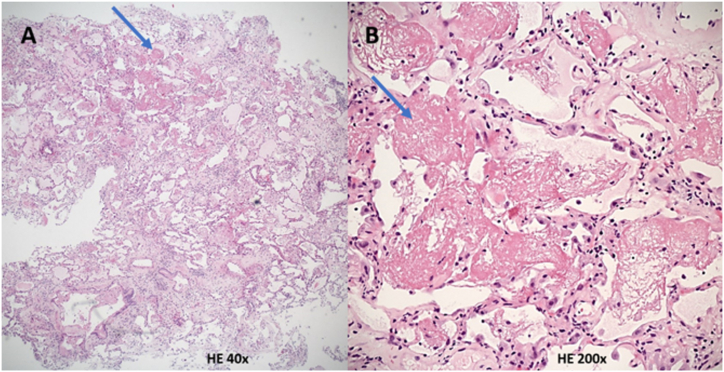
**Histopathological analysis of lung cryobiopsies.** HE = Hematoxylin and Eosin coloration. Blue arrows show intra-alveolar fibrin deposition (“fibrin balls”).

Bronchoscopy was performed and showed neither purulent secretions nor the presence of pathogenic microorganisms. Broncho-alveolar lavage (BAL) cytology was consistent with an inflammatory process with 980′000 cells/ml, lymphocytosis and eosinophilia (52 % lymphocytes, 8 % eosinophils, 20 % neutrophils and 20 % macrophages). Transbronchial biopsies yielded inconclusive results (AxBx) and a negative C4d staining according to ISHLT 2007 Classification [3]. Donor-specific-antibodies (DSA) were negative in blood samples. A treatment with IV methylprednisolone 500 mg daily for 3 days was administered. After a relative clinical improvement allowing the patient to return home during three weeks, hypoxemia occurred with a SpO2 of 70 % on ambient air. The patient was re-admitted and a broad-spectrum antibiotic was introduced (Piperacillin-Tazobactam). Transbronchial lung cryobiopsies (TLCB) excluded acute cellular rejection (A0B0, negative C4d) but showed diffuse intra-alveolar fibroblastic plugs (“fibrin balls”) confirming the diagnosis of AFOP (Fig. 2). Both bronchial aspiration and BAL PCR were positive for *Aspergillus* spp (286′000 and 41′000 copies/ml, respectively) without hyphae at histopathology. Serial galactomannan and beta-D-glucan measurements in blood were negative.

TLCB were well tolerated but the patient developed acute severe hypercapnic respiratory failure necessitating orotracheal intubation (OTI) within 24 hours. Emergency bilateral lung retransplantation was discussed in a multi-disciplinary meeting but perioperative mortality risks were judged too high, notably due to severe malnutrition and sarcopenia. IV infliximab 250 mg (based on case reports [4,5]), tocilizumab 8mg/kg (based on IL-6 elevation in serum cytokinic panel [213 pg/ml for a normal value of <7.0 pg/ml]) and corticosteroids (prednisone 1 mg/kg) were administered. *Aspergillus* spp colonization was treated with IV voriconazole.

The patient's ventilatory parameters improved slowly, allowing extubation after 11 days. A second dose of infliximab 150 mg was administered 3 weeks later. A thoracic CT-scan performed one-month after AFOP diagnosis showed improvement of apical ground glass opacities (Fig. 1C and D). The patient was discharged home 42 days post-OTI with 1L/min O_2_. She presented important pleuritic pain needing opioid treatment.

Thoracic CT scan 3 months after diagnosis showed a near-complete resolution of ground glass opacities with stabilization of peribronchial condensations (Fig. 1E and F). The patient remained clinically stable at home during 118 days but developed severe pleuritic pain requiring hospitalization for analgesic treatment with fentanyl infusion pump. She had an episode of suspected bronchoaspiration and developed hypercapnic respiratory failure. The patient was transferred to the intensive care unit but refused noninvasive and invasive ventilation. In accordance with the patient's wishes, end-of-life care was administrated and she passed away 122 days post AFOP diagnosis. The whole clinical history is summarized in Table 1.

**Table 1:** Clinical course.

Days to AFOP diagnosis	Clinical course	Treatment
**−1963**		Bilateral lung transplantation for cystic fibrosis
**−92**	mMRC 2 dyspnea, no cough, no purulent expectoration	First consultation in the adult outpatient clinic
**−68**		Spirometry: FEV1 2.16 L, FVC 2.43 L, FEV1/FVC 0.89
**−36**	mMRC 3 dyspnea, dry cough and symptoms of upper respiratory infection	Nasopharyngeal swab PCR: Adenovirus and Rhinovirus Chest radiography: no sign of infection
**−33**		Levofloxacine 500 mg/day for 10 days
**−26**		Minocycline 100 mg 2 times daily for 4 days
**−23**		Spirometry: loss of 1.14 L of FEV1 for a total FEV1 of 1.04 L (34 % of the PV and 47 % of her BEST FEV1).
**−22**		Bronchoscopy: Cytology with 980′000 cells/ml and lymphocytosis (52 % lymphocytes, 8 % eosinophils, 20 % neutrophils and 20 % macrophages). Transbronchial biopsies: AxBx with negative C4d → First hospitalization with IV Methylprednisolone 500 mg daily for 3 days and introduction of Azithromycin
**−20**	mMRC 2 dyspnea with diminution of cough	Discharged home
**−7**	mMRC 4 dyspnea with bilateral basithoracic pain	
**−1**	Hypoxic respiratory failure with SpO2 70 % on ambient air	Thoracic CT: panlobar multifocal ground glass opacities bilaterally and peribronchial condensations with a predominance in lower lobes
**0**		Cryobiopsies: diffuse intra-alveolar fibroblastic plugs (“fibrin balls”). A0B0. BAL PCR positive for *Aspergillus fumigatus* Piperacilline-Tazobactam for 7 days
**+1**	Severe global respiratory failure	Transfer to the ICU for non-invasive ventilation IV infliximab 250 mg IV voriconazole
**+2**	Severe global respiratory failure	Oro-tracheal intubation
**+6**		IV tocilizumab (8 mg/kg)
**+12**		Extubation
**+23**		IV infliximab 150 mg
**+ 44**	mMRC 3 dyspnea with dry cough	Discharged home with 1 L/min O2
**+ 98**	mMRC 2–3 dyspnea	Thoracic CT: regression of ground-glass opacities with stabilization of peribronchial condensations
**+118**	Increasing pleuritic pain	Hospitalization for antalgic treatment with Fentanyl intravenous infusion. Possible bronchoaspiration Development of severe hypercapnia
**+122**	Development of hypercapnic respiratory failure	End of life care leading to death

## 3 Discussion

The first definition of AFOP in 2002 by *Beasley* et al. was made after the identification of specific histology patterns in 17 lung specimens from non-transplant patients with clinical acute lung injury. Compared to the well documented histology of diffuse alveolar damage or bronchiolitis obliterans with organizing pneumonia, AFOP is characterized predominantly by intra-alveolar fibrin and organizing pneumonia [1]. With a follow-up of 1.1 year, the authors described 2 distinct patterns of disease progression with either fulminant-illness leading to death or subacute presentation with recovery.

Since then, AFOP has been described in several case reports and case series. In the last classification update by the American Thoracic Society and European Respiratory Society, it was recognized as a rare type of idiopathic interstitial pneumonia [6]. The exact etiology of AFOP remains unknown. Various clinical conditions seem to be associated with AFOP development, the most prevalent being infectious triggers, autoimmune diseases, drugs or environmental causes [7]. While AFOP has been described in allogenic hematopoietic stem cell transplant [4,8,9], lung transplantation seems to be an independent risk factor with almost 100 cases described in the literature (Table 2).

**Table 2:** Studies reporting acute fibrinous and organizing pneumonia in lung transplant recipients.

Study	Number of cases	Age	CT findings	Anti-microbial treatment	Immunosuppressive treatment	Evolution	Time from onset to death
Paraskeva et al., 2013	22	40 (IQR: 29–56)	Ground glass (91 %) Interlobular septal thickening (91 %) Consolidations (23 %) Peripheral fibrosis (23 %)	100 % broad spectrum antibiotic Oseltamivir (60 %) Gangyclovir IV (>95 %)	Methylprednisolone 5 mg/kg for 3 days (50 %)	95.5 % death (21/22) during the study period	Median 101 days
Vanstapel et al., 2020	54^a^	N/A	Ground-glass opacities (69 %) Consolidations (48 %) Nodular opacifications (33 %)	N/A	Plasmapheresis and IVIG (19 %) Methylprednisolone 500 mg/day for 3 days (24 %) Rituximab (9 %)	N/A	N/A
Renaud-Picard et al., 2015	1	22	Extensive ground glass opacities, nodules and micronodules Traction bronchiectasis	IV Ceftazidime Oral Levofloxacin IV Caspofungine IV Valganciclovir Sulfamethoxazole-trimethroprim	Methylprednisolone 750 mg/day for 3 days	Retransplantation and survival	–
Otto et el. 2013	1	66	Diffuse ground-glass opacities Lower lobes consolidation Bronchiectasis	Meropenem Clarithomycin Oseltamivir Zanamivir	High-dose corticosteroids	Death	9 days
Alici et al., 2015	1	48	Diffuse ground-glass opacities Lower lobes consolidations Interlobular septal thickening	Broad spectrum antibiotic IV antigungal therapy IV antiviral therpay	Methylprednisolone 1g for 3 days	Survival	–
Meyer et al., 2015	6	26–71	Diffuse ground-glass opacities Perilobular consolidations	Antibiotics (100 %)	Corticosteroids (100 %)	5/6 death due to AFOP	60 45 222 160 672

a In this paper, early (<90 days post-transplant) and late (>90 days post-transplant) AFOP were analyzed separately. The numbers shown in this table represent early and late AFOP grouped together.

**Fig. 3:**
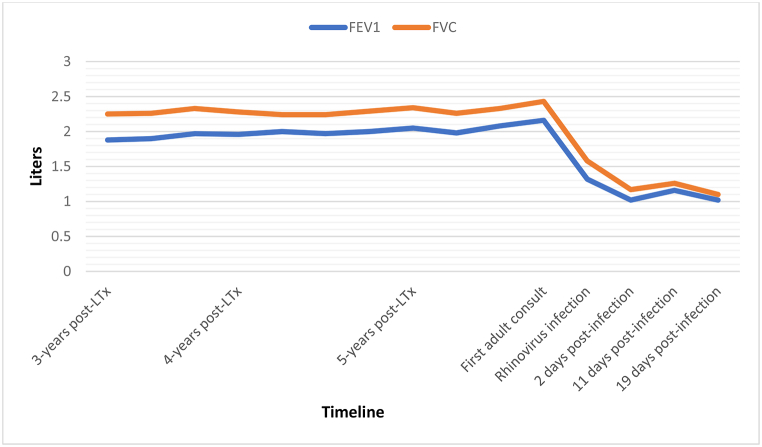
**Evolution of pulmonary function tests.** Harmonious decrease of forced expiratory volume in the first second (FEV1) and forced vital capacity (FVC) post viral infection.

In a retrospective study of 194 bilateral LT published in 2013, *Paraskeva* et al. proposed to classify AFOP as a new phenotype of chronic lung allograft dysfunction (CLAD), different from the known forms of bronchiolitis obliterans syndrome (BOS) and restrictive allograft syndrome (RAS) [2]. Interestingly, in 2020, *Vanstapel* et al. described a possible link between AFOP and RAS, making AFOP a possible subtype of RAS instead of a new entity of CLAD [10,11]. To date, there is no final consensus on this subject. In our case report, patient's pulmonary function tests showed a harmonious FEV1 and FVC decrease without obstructive syndrome (FEV1/FVC = 0,83) suggesting a restrictive pattern (Fig. 3).

Our patient initially presented with dyspnea and dry cough, consistent with symptoms described in the literature, the most common being fever, cough, dyspnea and sputum production [12]. We identified 2 potential risk factors for AFOP: First, a poor adherence to immunosuppressive therapy (one blood analysis 2 months before diagnosis showing non-measurable tacrolimus levels). Low levels of tacrolimus have been described in a case report of AFOP in a LT recipient [13] and the use of tacrolimus was associated with a lower risk of AFOP development [2]. Second, respiratory viral infections have been associated with AFOP [7]. The trigger in our case was identified with a positive NPS for Adenovirus and Rhinovirus. While *Aspergillus* spp may also be linked to AFOP development [7], it has been described in hematological patients with prolonged agranulocytosis and a risk for pulmonary fungal invasive disease [14]. We believe that Aspergillus identification in our patient was incidental and suggestive of colonization, considering the absence of radiological or biological elements for invasive pulmonary aspergillosis. Targeted therapy for *Aspergillus* spp was however preventively administered due to the immunosuppressive treatment introduced for AFOP.

As the first symptoms of AFOP usually mimic infection or rejection and due to its low prevalence, diagnosis could be delayed. The dominant radiologic pattern is diffuse or patchy distribution of ground-glass opacities and consolidations [15], highlighting the difficulty to distinguish acquired infections from AFOP [12]. This was also the case for our patient when we first tried to treat an opportunistic lung infection. In the absence of clinical improvement, acute cellular rejection was suspected and the patient received high-dose corticosteroids. Blood analysis did not reveal DSA and TLCB did not show cellular rejection (A0B0). Previous reports describe a good diagnostic yield of TBLC for AFOP compared to conventional transbronchial biopsies, attributed to the larger sample size. Other authors support that TBLC may be a safe and effective modality for acutely ill-hospitalized patients with diffuse pulmonary lung disease. It seems to be safer than surgical lung biopsy (SLB) in this kind of non-elective and emergency situation. In this case, while radiologic pattern was consistent with AFOP, final diagnosis was made only after identification of diffuse intra-alveolar fibrin on TBLC histology. In the literature, 100 % of LT recipients suffering from AFOP received high-dose corticosteroids (Table 2) and some cases reported a successful treatment without other concomitant immunosuppressive therapy [16,17].

In our case, after 19 days of improvement, the patient developed severe respiratory failure requiring hospitalization in the intensive care unit (ICU) and mechanical ventilation where high-dose corticosteroids were readministered. Our patient received infliximab, a monoclonal antibody with anti TNF-α activity. This decision for this treatment was made after a review of the literature showing expression of HO-1 and TNF-α in AFOP lung specimens [4,5], with one case report describing successful treatment with an anti TNF-α therapy [4]. This therapy however has never been reported in LT recipients.

In our patient, serum cytokine panel showed a mild interleukin-6 (IL-6) elevation. This finding was used to guide treatment by an IL-6 inhibitor (Tocilizumab). Unfortunately, cytokine panel in bronchoalveolar lavage (BAL) was not available due to a technical issue. To our knowledge, IL-6 inhibitor treatment has never been used in AFOP before. Finally, while one case of successful AFOP treatment with retransplantation was reported [13], in our patient, the mortality risk was judged too high. The mechanical ventilation mode was compatible with a severe restrictive pattern with decreased compliance and high driving pressure despite the use of protective ventilation settings at 4 ml/kg of predicted body weight. Extracorporeal membrane oxygenation was not used and the patient lung function finally improved allowing extubation after 12 days. A second dose of TNF-α inhibitor was given 11 days post extubation, without any side effect and oral high-dose corticosteroids (prednisone 1 mg/kg) was given until the discharge home, with subsequent slow tapering. On the respiratory level, the patient remained stable at home during 4 months. She however developed severe pleuritic pain, a symptom that has been described in a previous series of AFOP patients to be as frequent (up to 30.8 %) [18]. She required increasing doses of opioids, leading to severe hypercapnia and eventually to death.

Our case firstly described an initial favorable course of AFOP in a LT recipient treated with high-dose systemic corticosteroids, anti-TNF-α and anti-IL-6 target therapies. Cytokine pattern measurement in blood and BAL may help therapeutic decisions in this dramatic clinical condition, but this should be validated prospectively.

## 4 Conclusion

Acute fibrinous and organizing pneumonia (AFOP) is a rare yet severe complication after lung transplantation, frequently leading to an urgent evaluation for re-transplantation. While high-dose systemic corticosteroids remain the first line of treatment, the use of anti TNF-α has shown promising results in case reports. Furthermore, we propose prompt realization of a cytokine panel analysis in both blood and bronchoalveolar lavage to better guide the adjuvant administration of a targeted anti-inflammatory therapy.

## CRediT authorship contribution statement

**Christophe Abellan:** Conceptualization, Data curation, Investigation, Methodology, Writing – original draft, Writing – review & editing. **Foteini Ioakeim:** Investigation, Methodology, Writing – original draft. **Alessio Casutt:** Investigation, Methodology, Project administration, Writing – review & editing. **Benoit Lechartier:** Writing – review & editing. **Zisis Balmpouzis:** Writing – review & editing. **Samuel Rotman:** Resources, Writing – review & editing. **Leslie Noirez:** Writing – review & editing. **Isabelle Rochat:** Writing – review & editing. **John-David Aubert:** Writing – review & editing. **Angela Koutsokera:** Conceptualization, Project administration, Supervision, Writing – review & editing.

## Funding sources

This study was not supported by any sponsor or funder.

## Declaration of competing interest

The authors declare that they have no known competing financial interests or personal relationships that could have appeared to influence the work reported in this paper.
